# Optimization and Characterization of Microwave-Assisted Hydro-Distillation Extraction of Essential Oils from *Cinnamomum camphora* Leaf and Recovery of Polyphenols from Extract Fluid

**DOI:** 10.3390/molecules25143213

**Published:** 2020-07-14

**Authors:** Ao Shang, Ren-You Gan, Jia-Rong Zhang, Xiao-Yu Xu, Min Luo, Hong-Yan Liu, Hua-Bin Li

**Affiliations:** 1 Guangdong Provincial Key Laboratory of Food, Nutrition and Health, Department of Nutrition, School of Public Health, Sun Yat-Sen University, Guangzhou 510080, China; shangao@mail2.sysu.edu.cn (A.S.); xuxy53@mail2.sysu.edu.cn (X.-Y.X.); luom65@mail2.sysu.edu.cn (M.L.); 2 Research Center for Plants and Human Health, Institute of Urban Agriculture, Chinese Academy of Agricultural Sciences, Chengdu 610213, China; ganrenyou@caas.cn (R.-Y.G.); liuhongyan01@caas.cn (H.-Y.L.); 3 Department of Food Science & Technology, School of Agriculture and Biology, Shanghai Jiao Tong University, Shanghai 200240, China; zhangjiarong@sjtu.edu.cn

**Keywords:** *Cinnamomum camphora*, microwave-assisted extraction, essential oil, polyphenols, response surface methodology, GC×GC-TOFMS

## Abstract

In this study, the efficiency of microwave-assisted hydro-distillation (MAHD) to extract essential oil from *Cinnamomum camphora* leaf, and the recovery of polyphenols from extract fluid were investigated. The effects of microwave power, liquid-to-material ratio, and extraction time on the extraction efficiency were studied by a single factor test as well as the response surface methodology (RSM) based on the central composite design method. The optimal extraction conditions were a microwave power of 786.27 W, liquid-to-material ratio of 7.47:1 mL/g, and extraction time of 35.57 min. The yield of essential oil was 3.26 ± 0.05% (*w/w*), and the recovery of polyphenols was 4.97 ± 0.02 mg gallic acid equivalent/g dry weight under the optimal conditions. Furthermore, the comprehensive two-dimensional gas chromatography-time-of-flight mass spectrometry (GC×GC-TOFMS) was used to characterize the essential oils of fresh and fallen leaves, and 159 individual compounds were tentatively identified, accounting for more than 89.68 and 87.88% of the total contents, respectively. The main ingredients include sabinene, l-β-pinene, β-myrcene, α-terpineol, 3-heptanone, and β-thujene, as well as δ-terpineol and 3-heptanone, which were first identified in *C. camphora* essential oil. In conclusion, the MAHD method could extract essential oil from *C. camphora* with high efficiency, and the polyphenols could be obtained from the extract fluid at the same time, improving the utilization of *C. camphora* leaf.

## 1. Introduction

The *Cinnamomum camphora* is known as an evergreen tree of Lauraceae, widely distributed in China and Japan [1]. The *C. camphora* is not only a high-quality resource of wood but also plays an important role in the utilization of fragrance, medicine, and chemical industry due to its special aromatic and medicinal value [1,2,3,4]. In addition, some studies indicated that the extract of *C. camphora* leaf and its bioactive components have antioxidant, anti-inflammatory, antialgal, antimicrobial, and insecticidal activities in vitro, as well as the effects of an anticonvulsant, neuroprotective, and alleviating atopic dermatitis in vivo [2,5,6,7].

*C. camphora* is a good resource of essential oil. The essential oil of *C. camphora* is an important material for perfume, and it also has a variety of bioactivities [8,9]. According to the literature, the *C. camphora* essential oil has an antigenotoxic effect, such as inducing mitochondrial dysfunction and reducing nuclear mutations [10]. In addition, the antimicrobial activity of *C. camphora* essential oil has received wide attention, such as the antibacterial activity against *Bacillus subtilis*, *Enterococcus faecalis*, *Escherichia coli*, *Pseudomonas aeruginosa*, *Staphylococcus aureus*, and *Salmonella gallinarum*, as well as the antifungal effect against *Alternaria solani* Sorauer, *Gacumanomyces graminis*, and *Choanephora cucurbitarum* [11,12,13,14,15]. Moreover, the essential oils of *C. camphora* have high insecticidal and repellent activities against *Anopheles stephensi*, *Aphis gossypii*, *Tribolium castaneum*, and *Lasioderma serricorne* [9,16]. Therefore, *C. camphora* essential oil has high application value and broad development prospects.

At present, steam distillation is the most common and convenient method to extract essential oils from plants, but the whole process is time-consuming and relatively inefficient [17]. Due to the high efficiency, microwave-assisted extraction of plant essential oils has become a focused area of research [18]. Microwave radiation can destroy cell walls by swelling cells, changing the intracellular structure, breaking glands and oil-rich cells, and accelerating the motion of aqueous solution and the diffusion of internal ingredients [19]. Microwave-assisted hydro-distillation (MAHD) extraction with water as a solvent is a green and environmentally friendly method, which can be used for the extraction of plant essential oils [20,21,22]. In the process of MAHD, the extraction efficiency is affected by several parameters, such as the microwave power, liquid-to-material ratio, and extraction time [20,21,23]. These parameters have a direct influence on the extraction efficiency, and they also interact with each other. Hence, the response surface methodology (RSM) can be adopted to analyze and optimize extraction conditions and obtain a fitting model [23]. On the other hand, the comprehensive two-dimensional gas chromatography-time-of-flight mass spectrometry (GC×GC-TOFMS) can be used to separate complex mixtures, and has been successfully applied to analyze the volatile components of essential oils [24,25,26]. GC×GC-TOFMS performs the two-dimensional separation of compounds by combining two coupled columns, which greatly improves the peak capacity and resolution as well as the sensitivity [27,28].

In this paper, an MAHD method was established to extract the essential oil from *C. camphora* fresh leaf, and recover polyphenols from extract fluid. The single factor experiments and response surface methodology (RSM) were used to optimize the extraction conditions. In order to fully utilizing forest waste resources, the application of the obtained optimal extraction conditions to *C. camphora* fallen leaf was also discussed. Additionally, the chemical compositions of *C. camphora* essential oil were isolated and identified by GC×GC-TOFMS.

## 2. Results and Discussion

### 2.1. Analysis of Single-Factor Experiments

#### 2.1.1. Effect of Microwave Power

**Figure 1:**
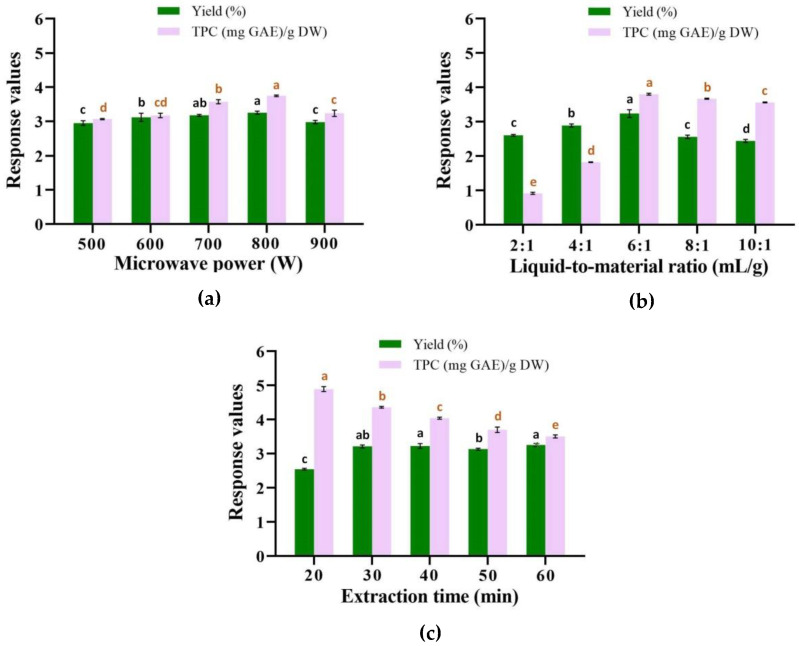
Effects of single factors on the yield of essential oil and content of total polyphenols in extract fluid: microwave power (**a**), liquid-to-material ratio (**b**), and extraction time (**c**). Different letters within a, b, c, and d in black color indicate significant differences (*p* < 0.05) in the essential oil yield among groups; Different letters within a, b, c, d, and e in red color indicate significant differences (*p* < 0.05) in the total phenol content (TPC) among groups. The same letter indicates no significant difference (*p* > 0.05) among groups. The significant differences among groups were analyzed by one-way ANOVA with Duncan *post hoc* test at *p* < 0.05.

Microwave power is an important influencing factor in the microwave-assisted extraction process. The other conditions were fixed on the liquid-to-material ratio of 6:1 mL/g and the extraction time of 60 min, and the effects of microwave power at 500, 600, 700, 800, and 900 W on the yield of fresh leaf essential oil and the total phenol content (TPC) in the extract fluid were examined, as shown in Figure 1a. According to the experimental results, the essential oil yield increased slightly when the microwave power was 500–800 W, reached the highest at 800 W, and then decreased significantly at 900 W. With the increase of microwave power, the polarization of polar molecules caused by microwave is enhanced, and the heat motion of molecules as well as the cell rupture is promoted, thereby the output of essential oil is increased [29]. On the other hand, excessive microwave power may lead to the decomposition of some thermo-sensitive metabolites, which affects the essential oil yield [18]. On the other hand, as the microwave power ranged from 600 to 800 W, the TPC gradually increased, reaching a peak at 800 W, and then decreasing at 900 W. The high microwave power could result in the degradation of phenolic compounds [30]. Considering the influence of microwave power on the essential oil yield and the TPC value, 800 W was chosen as the optimal microwave power.

#### 2.1.2. Effect of Liquid-to-Material Ratio

The ratio of liquid-to-material affects the efficiency of microwave extraction, and an appropriate liquid-to-material ratio can simultaneously ensure the extraction efficiency as well as saving production costs. In this section, the extraction efficiency of essential oil and TPC were investigated when the liquid-to-material ratio was set as 2:1, 4:1, 6:1, 8:1, and 10:1 mL/g, under the condition of 800 W and 60 min. The results (Figure 1b) showed that the yield of essential oil rose up with the increase of the liquid-to-material ratio from 2:1 to 6:1 mL/g, and attained the maximum at the ratio of 6:1 mL/g, and then gradually descended at 8:1 and 10:1 mL/g. When the liquid-to-material ratio is less, the contact between the solvent and sample is inadequate during the extraction process, leading to the lower output of essential oil. With the increase of the liquid-to-material ratio, the essential oil diffuses more in water, making it easier for vapor to carry out the essential oil, and the yield of essential oil increases. When the ratio continues to increase, the essential oil would be dissolved or emulsified by water, resulting in a decrease in the yield [31]. In addition, the variation trend of the TPC of the extract fluid was similar to that of the essential oil. The TPC value increased rapidly from the liquid-to-material ratio of 2:1 to 6:1 mL/g, and the maximum TPC value appeared at the ratio of 6:1 mL/g. From the liquid-to-material ratio of 6:1 to 10:1 mL/g, the TPC value declined slightly. The increase of solvent volume could promote the transfer of components to solution, improve the solubility, and elevate the extraction efficiency of polyphenols [32]. When the ratio of liquid-to-material is higher, the effect of increasing solvent is no longer significant, because most of the polyphenols have been dissolved, but this causes the consumption of solvent. Therefore, 6:1 mL/g was selected as the best liquid-to-material ratio for further optimization by RSM.

#### 2.1.3. Effect of Extraction Time

Under the condition of 800 W and 6:1 mL/g, the effects of an extraction time of 20, 30, 40, 50, and 60 min on the essential oil yield and TPC were studied, and the results are shown in Figure 1c. As the extraction time extended from 20 to 30 min, the essential oil yield obviously enhanced, and then the yield remained almost unchanged when the extraction time increased from 30 to 60 min. In addition, the TPC value also varied with the microwave time. When the microwave-assisted extraction was conducted for 20 min, the TPC value was the highest. With the extension of the extraction time, the TPC values gradually decreased. This was because the polyphenols might degrade during long-time microwave irradiation, resulting in a decrease of the TPC value [32]. Considering the effects of the extraction time on the essential oil yield and the TPC value, 30 min was set as the optimal value for RSM optimization.

### 2.2. Analysis of Response Surface Methodology

#### 2.2.1. Results of Central Composite Design (CCD)

According to the results of the single-factor experiments, the microwave power of 800 W, liquid-to-material ratio of 6:1 mL/g, and extraction time of 30 min were selected as the middle levels of CCD. The design of 20 runs and the corresponding response values, including the yield of fresh leaf essential oil and the TPC values of extract fluids, are shown in Table 1. The experiment results showed that the yield of essential oil ranged from 1.94% to 3.44% (*w*/*w*), and the TPC values of extract fluids ranged from 3.29 to 5.44 mg·GAE/g·DW.

#### 2.2.2. Model Fitting and Analysis of Variance (ANOVA)

The data of the CCD design were analyzed by multiple regression fitting, and the quadratic polynomial regression models for the essential oil yield and TPC value of extract fluid were established. Based on the ANOVA analysis, both models were statistically significant (*p* ≤ 0.0001) with F-values of 14.74 and 26.26, respectively. The determination coefficients (R^2^) were 0.9299 and 0.9594, both greater than 0.80, indicating that the model fitted well [33]. The adjusted R^2^ (R^2^_Adj_) was 0.8668 and 0.9299, which were close to their corresponding R^2^, further indicating that the fitting degree of the models was relatively high, and the predicted values had a high correlation with the actual values. Moreover, the high *p*-values of the “lack of fit” of the models were 0.2473 and 0.1399, suggesting that the “lack of fit” had no statistical significance, which verified the reliability of the models [34]. The variation coefficients (C.V.) of 5.75 and 3.54%, which were both lower than 10%, also indicated the high accuracy and predictability of the equations [35].

#### 2.2.3. Effects of Independent Variables on Essential Oil Yield

The results of the RSM experiments of *C. camphora* fresh leaf essential oil yield are shown in Table 1. Multiple regression fitting was carried out on the experimental data, and the ANOVA analysis of the model showed that the model was significant. The relationship between the three independent variables and the essential oil yield is described in the following coded regression equation (excluding insignificant coefficients):

Y_Yield_ = 3.31 + 0.32X_3_ + 0.24X_1_X_2_ − 0.10X_1_^2^ − 0.30X_2_^2^ − 0.19X_3_^2^, (1)

where Y refers to the yield of fresh leaf essential oil, X_1_ is the microwave power, X_2_ is the liquid-to-material ratio, and X_3_ is the extraction time.

Based on the results of the equation and ANOVA, the linear effect of the extraction time (X_3_) was positive and significant on the fresh leaf essential oil yield (*p* < 0.0001), and the cross-product effect between the microwave power and liquid-to-material ratio (X_1_X_2_) was also positive and significant (*p* < 0.01), while all the quadratic terms (X_1_^2^, X_2_^2^ and X_3_^2^) were negative and significant (*p* < 0.05).

**Figure 2:**
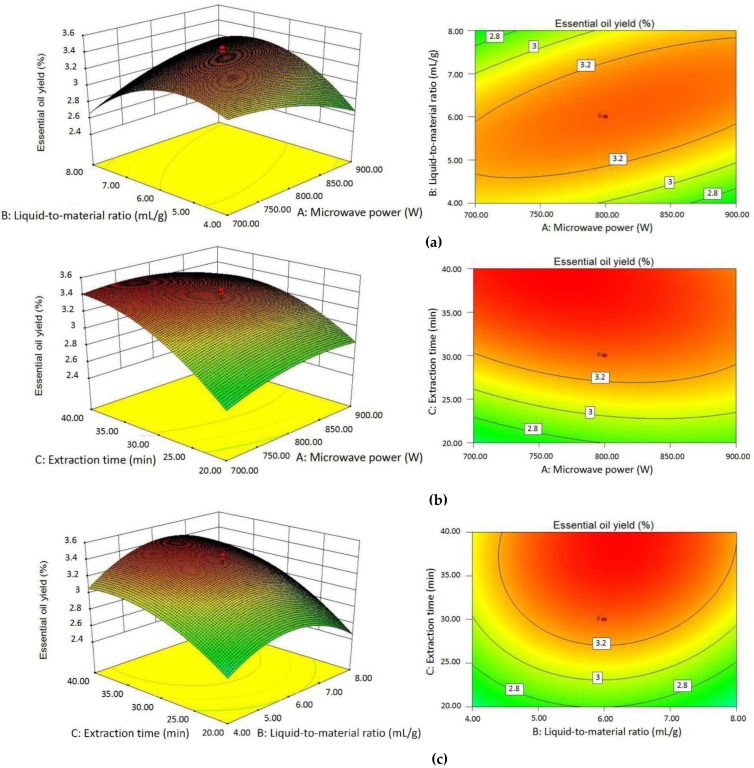
The three-dimensional response surfaces and contour plots of fresh leaf essential oil yield affected by microwave power and the liquid-to-material ratio (**a**); microwave power and extraction time (**b**); and liquid-to-material ratio and extraction time (**c**).

To visually illustrate the interaction of the independent variables on the yield of fresh leaf essential oil, the three-dimensional response surfaces and contour plots are shown in Figure 2a–c. Figure 2a displays the interaction between the microwave power and liquid-to-material ratio on the response values with a fixed extraction time of 30 min. It was observed that the yield of essential oil decreased slightly as the microwave power increased from 700 to 900 W, while the yield increased when the liquid-to-material ratio varied from 4:1 to 6:1 mL/g, and then decreased as the liquid-to-material ratio increased to 8:1. According to Figure 2b, the lower microwave power and longer extraction time were beneficial to elevate the essential oil yield at the liquid-to-material ratio of 6:1 mL/g. The essential oil yield markedly increased when the extraction time varied from 20 to 40 min. At a constant extraction time of 40 min, the influence of the microwave power on the essential oil yield was similar to that in Figure 2a. Figure 2c shows the response surface plot between the liquid-to-material ratio and the extraction time on the essential oil yield. Similar to Figure 2a,b, the extended time led to the increase of the essential oil yield, while with the increase of the liquid-to-material ratio, the yield of essential oil first rose and then declined. Combined with the results of ANOVA and the response surfaces above, the influences of the microwave power, liquid-to-material ratio, and extraction time on the yield of essential oil were mainly in a quadratic manner.

#### 2.2.4. Effects of Independent Variables on TPC Value

The RSM results of the TPC value of the extract fluid were measured by the Folin–Ciocalteu method, and the response values are shown in Table 1. The regression analysis was performed on the experimental data, and the relationship between the variables and TPC could be expressed in a coded equation (excluding insignificant coefficients):

Y_TPC_ = 4.65 − 0.22X_1_ + 0.57X_2_ − 0.13X_3_ − 0.15X_1_^2^ − 0.11X_3_^2^, (2)

where Y refers to the TPC value of the extract fluid, X_1_ is the microwave power, X_2_ is the liquid-to-material ratio, and X_3_ is the extraction time.

According to the ANOVA result, the linear terms of the microwave power (X_1_) and extraction time (X_3_) were both positive and significant for the TPC value (*p* < 0.05), while the effect of the liquid-to-material ratio (X_2_) was statistically reverse and very significant (*p* < 0.0001). Additionally, the microwave power and extraction time displayed significant negative quadratic effects (X_1_^2^ and X_3_^2^) on TPC (*p* < 0.05). All interaction terms (X_1_X_2_, X_1_X_3_, and X_2_X_3_) showed no significant influence (*p* > 0.05).

**Figure 3:**
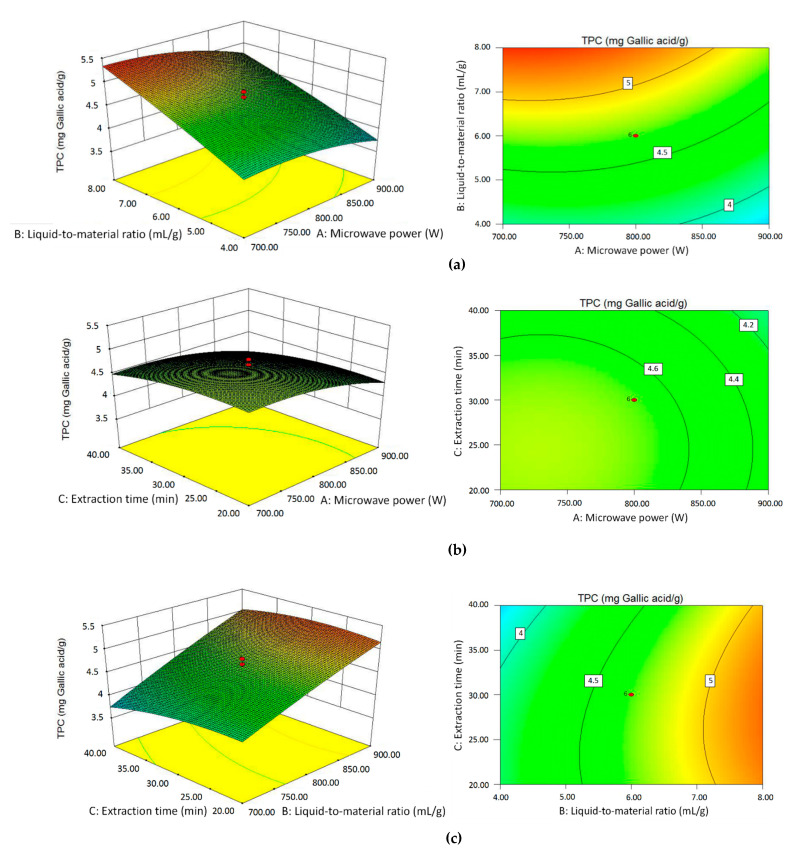
The three-dimensional response surfaces and contour plots of the TPC value affected by microwave power and the liquid-to-material ratio (**a**); microwave power and extraction time (**b**); and liquid-to-material ratio and extraction time (**c**).

The three-dimensional response surfaces and contour plots, as shown in Figure 3a–c, were obtained to illustrate the interaction of variables on the TPC value. In Figure 3a, when the extraction time was fixed at 30 min, with the increase of the microwave power, the TPC value first increased and then decreased, and the overall change was relatively stable. With the increase of the liquid-to-material ratio, the TPC value increased significantly. Figure 3b exhibits the interaction between the microwave power and extraction time on the TPC value at a liquid-to-material ratio of 6:1 mL/g. As the liquid-to-material ratio and extraction time increased, the TPC value elevated slightly first and then declined, suggesting the quadratic effect of the two parameters on the TPC value. In Figure 3c, the influences of the liquid-to-material ratio and extraction time on the TPC value at the microwave power of 800 W are similar to those in Figure 3a,b. From the three groups of response surface plots, the liquid-to-material ratio had the most significant effect on the TPC value compared with the microwave power and extraction time. Besides, the microwave power and extraction time had quadratic effects on the TPC value.

#### 2.2.5. Verification of the Predicted Value

The optimal extraction conditions of *C. camphora* fresh leaf essential oil and polyphenols in extract fluid obtained by RSM were as follows: Microwave power of 786.27 W, liquid-to-material ratio of 7.47:1 mL/g, and extraction time of 35.57 min. Under this optimal condition, the predicted yield of essential oil was 3.28% (*w*/*w*), and the predicted TPC value of the extract fluid was 5.04 mg·GAE/g·DW. The reliability of the model was determined by the verification experiment under the optimal conditions, and the yield of fresh leaf essential oil was 3.26 ± 0.05% (*w*/*w*) and the TPC value was 4.97 ± 0.02 mg·GAE/g·DW. The actual values were close to the predicted values, which proved the accuracy of the model. In addition, the same optimal extraction conditions were used to extract the essential oil of *C. camphora* fallen leaf, and the yield was 1.87 ± 0.06% (*w*/*w*), and the TPC value of extract fluid was 2.56 ± 0.04 mg GAE/g DW. The yield of essential oil extracted from *C. camphora* fresh leaf and fallen leaf under the optimal condition was significantly different, which might be due to the different essential oil composition of fresh leaf and fallen leaf.

### 2.3. Chemical Profile of C. camphora Essential Oils

The volatile compositions of essential oils from *C. camphora* fresh leaf and fallen leaf obtained by optimized microwave-assisted hydrodistillation extraction were analyzed by GC×GC-TOFMS. The qualitative analysis was performed by comparing with the mass spectrometry from the National Institute of Standards and Technology (NIST) mass spectrometry library, and the quantitative analysis was conducted by peak area normalization. A total of 159 compounds with a similarity of more than 800 and a peak area of more than 0.02% were identified, accounting for 89.68% and 87.88% of the total essential oil from *C. camphora* fresh leaf and fallen leaf, respectively, as shown in Table 2. To our knowledge, 109 of these compounds have not been previously identified in *C. camphora* leaf essential oil, mainly including δ-terpineol, (3-Methyl-oxiran-2-yl)-methanol, 3-heptanone, 2-[2-(ethenyloxy)ethoxy]-ethanol, formaldehyde, *cis*-sabinene hydrate, (+)-α-terpineol, 2-azido-2,3,3-trimethylbutane, ethanolamine, and (1-hydroxyethylidene)malonic acid diethyl ester [9,14,16,36,37,38].

**Figure 4:**
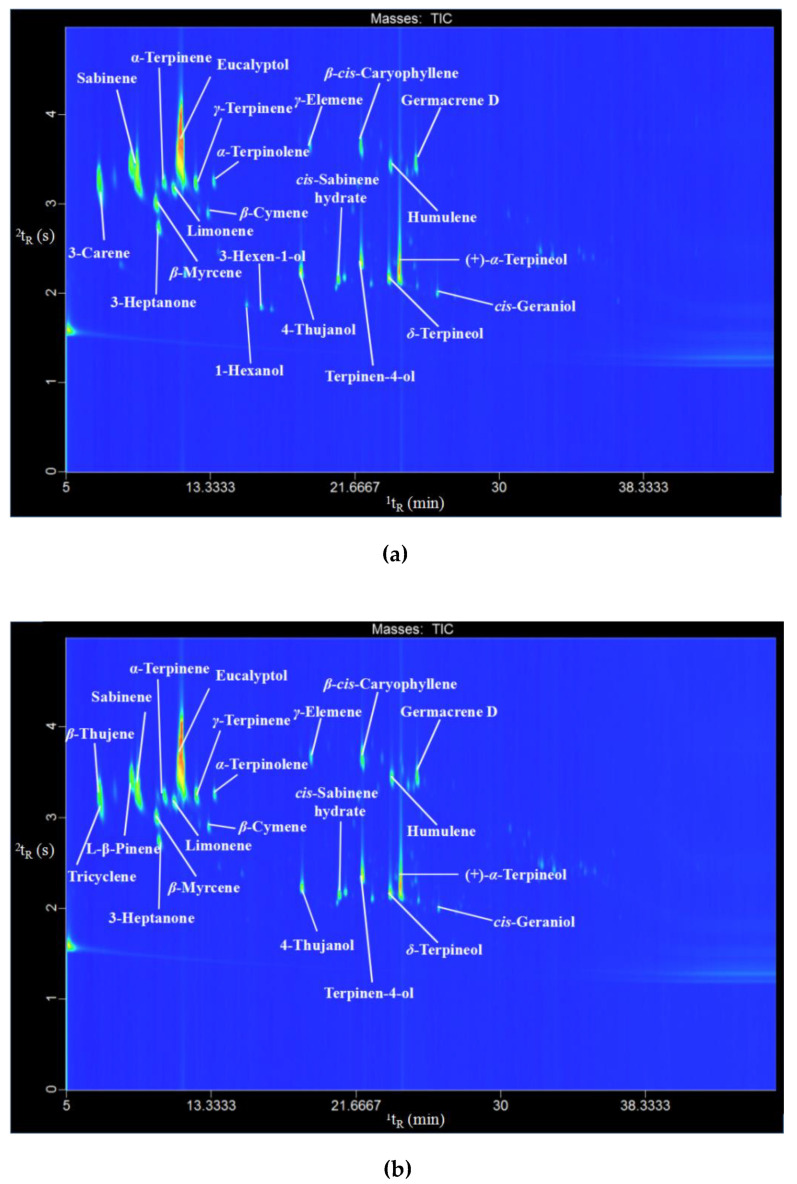
GC×GC total ion chromatogram (TIC) contour plot of two kinds of essential oils from fresh leaf (**a**) and fallen leaf (**b**).

According to Table 2, the chemical composition of essential oils of fresh and fallen leaves was similar, and there were 108 identical components in these two essential oils, but the main compounds were different. The major compounds in fresh leaf were sabinene, β-myrcene, δ-terpineol, 3-heptanone, and γ-terpinene, while the major compounds in fallen leaf were l-β-pinene, *α*-terpineol, δ-terpineol, β-thujene, and (3-methyl-oxiran-2-yl)-methanol. In addition, the GC×GC total ion chromatogram (TIC) contour plots of the essential oils of two kinds of leaves are shown in Figure 4, and the main compounds of *C. camphora* essential oil are marked. The composition of *C. camphora* essential oil might be influenced by variety, such as harvest time and maturity [9].

## 3. Materials and Methods

### 3.1. Plant and Reagents

Fresh and fallen leaves of *Cinnamomum camphora* were obtained from Sichuan province, China, and the plant was authenticated by Dr. Hong-Yan Liu, Research Center for Plants and Human Health, Institute of Urban Agriculture, Chinese Academy of Agricultural Sciences, Chengdu, Sichuan province, China. The leaves were washed, ventilated, and dried at room temperature (25 °C) for 1 day to dry the blade surface. The dried leaves were ground by a grinder (RS-FS500B, Royalstar Co., Ltd., Hefei, China), and sifted through a 40-mesh sieve (0.425 mm). The moisture content of the fresh leaf particles was 54.88 ± 0.04%, and that of fallen leaf particles was 30.22 ± 0.27%. The treated samples were stored at 4 °C for further experiments.

The chemicals, including gallic acid, anhydrous sodium carbonate, and Folin–Ciocalteu reagent, were of analytical grade and were purchased from Sigma-Aldrich St. Louis, MO, USA. Acetone, methanol, and *n*-hexane were of chromatographic grade and obtained from Macklin Chemical Factory (Shanghai, China). The deionized water was used in experiments.

### 3.2. Microwave-Assisted Hydro-Distillation (MAHD)

The MAHD device consists of a microwave oven (X-100A, Xianghu Instrumental Company, Beijing, China) and a Clevenger apparatus. The *C. camphora* leaf sample (20.0 g·DW) was placed in a distillation flask, and a certain amount of deionized water was added. The mixture was heated by microwave radiation, and the oil fraction was collected after distilling for a period of time. The anhydrous sodium sulfate was added for drying, and then the dried essential oil was weighed. The yield of essential oil (Y) was calculated as follows:

Y (%, *w/w*) = (dried essential oil mass/dried leaf sample mass) × 100. (3)

### 3.3. Total Phenolic Content Measured by the Folin–Ciocalteu Method

The total phenolic content was determined spectrophotometrically by the Folin–Ciocalteu method according to the literature with minor modification [39]. In brief, the 0.50-mL diluted sample was added to 2.5 mL of 0.2 mol/L Folin–Ciocalteu reagent, and thoroughly mixed. The mixture was incubated for 4 min, then 2 mL of saturated sodium carbonate solution were added. The absorbance of the mixture was determined at 760 nm after incubation shielded from light at room temperature (25 °C) for 2 h. Gallic acid was used as a reference standard, and the results were expressed as mg gallic acid equivalent (GAE)/g dry weight (DW) of leaf samples.

### 3.4. Single-Factor Experiment Design

The single-factor experiment was used to preliminarily optimize the microwave radiation power, liquid-to-material ratio, and extraction time, which are key factors influencing the extraction efficiency. The experimental design of each factor at each level is shown in Table 3.

### 3.5. Response Surface Methodology (RSM) Design

Based on the results of the single-factor experiment, a three-factor five-level response surface analysis based on the central composite design (CCD) was used to optimize the extraction conditions. The RSM design was conducted by Design Expert V8.0.6, with fewer iterations and higher efficiency. The experimental factors and levels are shown in Table 4.

In this CCD, 20 different experiments with 6 repetitions of the center points were carried out to fit the following quadratic equation:

(4) $$Y=\beta_{0}+\sum_{i=1}^{3}\beta_{i}x_{i}+\sum_{i=1}^{3}\beta_{\mathrm{ii}}x_{i}^{2}+\sum_{i=1}^{3}{\sum_{j>i}^{3}\beta_{\mathrm{ij}}x_{i}x_{j}}_{},$$

where Y is the predicted response value (essential oil yield or TPC value); β_0_ is the intercept of the equation; β_i_, β_ii_, and β_ij_ indicate the coefficients of linear, quadratic, and interaction terms; and x_i_ and x_j_ represent different independent variables (microwave power, liquid-to-material ratio, and extraction time).

The mathematical model of extraction conditions of essential oils and polyphenols was analyzed by analysis of variance (ANOVA) to test the adequacy of the equation, and the regression coefficients of each factor were tested. In addition, the three-dimensional surface plots and contour graphs were used to visualize the independence and interaction of each variable on the response values. The optimal conditions were executed, and the results were compared with the predicted values to determine the accuracy of the model.

### 3.6. GC×GC-TOFMS Analysis

The essential oil samples from fresh leaf and fallen leaf of *C. camphora* were diluted with n-hexane to obtain a solution of a 1% concentration, respectively. The GC×GC-TOFMS analysis was conducted on an Agilent 7890A gas chromatograph (Agilent Technologies, Santa Clara, CA, USA), connected to a Pegasus 4D time-of-flight mass spectrometer (LECO Corporation, St. Joseph, MI, USA). The GC×GC system consisted of two chromatographic columns in series, and the parameters of these two columns are shown in Table 5. The initial temperature of the first column was set at 60 °C (5 min hold) and then ramped to 240 °C at a rate of 5 °C/min (5 min hold). The secondary oven was maintained 5 °C offset above the primary oven. The carrier gas was helium with a purity of 99.9995%. The injector temperature was maintained at 250 °C. The injection volume was 1 μL, and was performed in the split mode with a split ratio of 1:5.

The mass spectrometer scanned in the range of *m/z* 28–600. The electron impact ionization energy was set at 70 eV. A 5-min solvent delay was conducted to protect the filament from solvent exposure.

All the operations and analysis of data were processed using LECO ChromaTOF^®^ software. The compositions of oil were identified by comparing the mass spectra with the National Institute of Standards and Technology (NIST) library data. The qualitative results of all compounds were given, including the compound name, Chemical Abstracts Service (CAS) registry number, similarity, retention time, and peak area. The similarity with the database is 1000 units, with the higher the similarity, the better the match. The relative abundance (%) was obtained by the ratio of the peak area of each compound to the sum of the peak areas of all compounds.

### 3.7. Statistical Analysis

All experiments were carried out in triplicate, and the results were expressed as mean ± standard deviation. The statistical analysis was performed using Design Expert V8.0.6 software (Stat-Ease Inc., Minneapolis, MN, USA), and SPSS 26.0 statistics software (IBM Corp., Armonk, NY, USA). The experimental data were statistically analyzed by one-way ANOVA with Duncan post hoc multiple comparisons test, and the significance level was set at *p* < 0.05.

## 4. Conclusions

A microwave-assisted hydro-distillation (MAHD) method was developed for the extraction of essential oils from *C. camphora* fresh leaf, and recovery of polyphenols from extract fluid. The response surface methodology (RSM) was used to optimize the experimental variables, including the microwave power, liquid-to-material ratio, and extraction time. The fitting models obtained by RSM were proved reliable. The optimal extraction conditions were a microwave power of 786.27 W, liquid-to-material ratio of 7.47:1 mL/g, and extraction time of 35.57 min. Under the optimal conditions, the essential oil yield and TPC value were 3.26 ± 0.05% (*w*/*w*) and 4.97 ± 0.02 mg GAE/g·DW, respectively. The microwave-assisted extraction could significantly shorten the extraction time and improve the extraction efficiency. In addition, GC×GC-TOFMS was used to analyze the volatile compounds of *C. camphora* essential oils from fresh leaf and fallen leaves. The essential oils of these two leaves differed in their chemical profile, and there were 108 common compounds in a total of 159 ingredients of the two leaves. The different composition of fresh leaf and fallen leaves might be influenced by the maturity of the leaves. The main components of fresh leaf were sabinene, β-myrcene, δ-terpineol, 3-heptanone, and γ-terpinene, while l-β-pinene, α-terpineol, δ-terpineol, β-thujene, and (3-methyl-oxiran-2-yl)-methanol were mainly found in the fallen leaf essential oils. Among them, δ-terpineol, 3-heptanone, and (3-methyl-oxiran-2-yl)-methanol were first identified in *C. camphora* leaf essential oil. Therefore, MAHD extraction is an interesting technology for the industrial production of *C. camphora* essential oil.

## Figures and Tables

**Table 1:** The experimental design and response values of central composite design (CCD).

Run	X_1_	X_2_	X_3_	Yield (%, *w*/*w*)	TPC (mg·GAE/g·DW)
	Microwave Power (W)	Liquid-to-Material Ratio (mL/g)	Extraction Time (min)		
1	700 (−1)	8:1 (1)	20 (−1)	1.94 ± 0.05	5.44 ± 0.03
2	631.82 (−1.68)	6:1 (0)	30 (0)	3.17 ± 0.12	4.51 ± 0.12
3	800 (0)	9.36:1 (1.68)	30 (0)	2.39 ± 0.08	5.44 ± 0.12
4	800 (0)	6:1 (0)	46.82 (1.68)	3.30 ± 0.24	4.32 ± 0.05
5	968.18 (1.68)	6:1 (0)	30 (0)	2.98 ± 0.12	3.98 ± 0.29
6	800 (0)	6:1 (0)	30 (0)	3.21 ± 0.21	4.81 ± 0.03
7	900 (1)	4:1 (−1)	40 (1)	2.62 ± 0.09	3.29 ± 0.31
8	900 (1)	8:1 (1)	20 (−1)	2.76 ± 0.18	4.81 ± 0.02
9	800 (0)	6:1 (0)	30 (0)	3.44 ± 0.14	4.53 ± 0.04
10	900 (1)	4:1 (−1)	20 (−1)	2.22 ± 0.05	3.82 ± 0.17
11	800 (0)	2.64:1 (−1.68)	30 (0)	2.63 ± 0.37	3.71 ± 0.13
12	900 (1)	8:1 (1)	40 (1)	3.35 ± 0.12	4.53 ± 0.05
13	700 (−1)	8:1 (1)	40 (1)	2.94 ± 0.05	5.18 ± 0.13
14	800 (0)	6:1 (0)	30 (0)	3.45 ± 0.09	4.60 ± 0.12
15	800 (0)	6:1 (0)	30 (0)	3.40 ± 0.18	4.64 ± 0.02
16	800 (0)	6:1 (0)	30 (0)	3.20 ± 0.07	4.81 ± 0.06
17	700 (−1)	4:1 (−1)	20 (−1)	2.37 ± 0.10	4.25 ± 0.10
18	700 (−1)	4:1 (−1)	40 (1)	3.14 ± 016	3.67 ± 0.02
19	800 (0)	6:1 (0)	13.18 (−1.68)	2.37 ± 0.09	4.39 ± 0.14
20	800 (0)	6:1 (0)	30 (0)	3.14 ± 0.03	4.69 ± 0.04

**Table 2:** Chemical profile identified from the essential oils of *C. camphora* fresh leaf and fallen leaf.

No.	Name	CAS	^1^ t_R_ (min)	^2^ t_R_ (s)	Fresh Leaf	Fallen Leaf
					Peak Area (%)	Peak Area (%)
1	2,4-Dimethyl-pentane	108-08-7	5.42	1.58	0.02–0.1	0.02–0.1
2	2,2,4-Trimethyl-oxetane	23120-44-7	5.42	2.18	0.02–0.1	0.02–0.1
3	Isopropyl peroxide	16642-57-2	5.58	1.58	0.02–0.1	-
4	2-Azido-2,3,3-trimethylbutane	51677-41-9	5.67	1.54	0.1–1.0	0.1–1.0
5	γ-Acetylpropyl acetate	5185-97-7	5.75	1.58	0.02–0.1	0.1–1.0
6	2-Ethylfuran	3208-16-0	5.75	2.02	0.02–0.1	<0.02
7	3-Acetyl-1-chloro-1,1,2,2-hexanetetracarbonitrile	-	6.17	2.04	-	0.02–0.1
8	(−)-α-Pinene	7785-26-4	6.83	3.26	0.1–1.0	<0.02
9	β-Thujene	28634-89-1	6.92	3.18	-	>1.0
10	Tricyclene	508-32-7	6.92	3.3	-	>1.0
11	3-Carene	13466-78-9	6.92	3.3	>1.0	-
12	β-*cis*-Ocimene	3338-55-4	7	3.22	0.1–1.0	-
13	Camphene	79-92-5	7.83	3.28	0.1–1.0	0.1–1.0
14	Hexanal	66-25-1	8.17	2.32	0.1–1.0	0.02–0.1
15	3,5,6-Trichloro-4-isopropylsulfanyl-pyridine-2-carbonitrile	216242-35-2	8.25	1.52	0.02–0.1	-
16	l-β-Pinene	18172-67-3	8.67	3.46	-	>1.0
17	β-Pinene	127-91-3	8.83	3.48	0.02–0.1	0.02–0.1
18	Sabinene	3387-41-5	9.08	3.46	>1.0	>1.0
19	β-Phellandrene	555-10-2	9.17	3.28	0.02–0.1	-
20	*o*-Methoxy-α-phenethylamine	-	9.25	3.2	0.1–1.0	-
21	4,5,6,7-Tetrahydro-5-benzyl-pyrrolo [3,2-c]pyridine	272442-27-0	9.33	3.14	0.1–1.0	-
22	*o*-Xylene	95-47-6	9.58	2.54	0.02–0.1	0.02–0.1
23	β-Myrcene	123-35-3	10.17	3.02	>1.0	>1.0
24	α-Phellandrene	99-83-2	10.25	3.24	0.02–0.1	0.1–1.0
25	3-Heptanone	13019-20-0	10.33	2.74	>1.0	>1.0
26	2,2-Dimethylpentanoic acid ethenyl ester	44970-05-0	10.5	3.2	0.02–0.1	0.02–0.1
27	α-Terpinene	99-86-5	10.67	3.26	>1.0	3.22
28	Methyl caproate	106-70-7	10.83	2.58	0.02–0.1	<0.02
29	3,7-Bis(ethylidene)-bicyclo [3.3.0]octane	-	10.92	2.76	0.02–0.1	-
30	Dehydro-1,8-cineole	92760-25-3	10.92	3.14	0.1–1.0	0.1–1.0
31	Limonene	138-86-3	11.25	3.18	>1.0	>1.0
32	5,6-Dimethyl-1,3-cyclohexadiene	5715-27-5	11.42	3.38	0.02–0.1	-
33	2,4-Dimethoxyaniline	2735-04-8	11.42	3.5	-	0.02–0.1
34	Boldenone sulfate	87331-43-9	11.67	4.12	<0.02	0.02–0.1
35	2-Hexenal	505-57-7	11.83	2.22	0.1–1.0	0.1–1.0
36	Eucalyptol	470-82-6	11.83	3.28	>1.0	0.1–1.0
37	α-Pinene	80-56-8	12.17	3	-	0.02–0.1
38	γ-Terpinene	99-85-4	12.5	3.26	>1.0	>1.0
39	β-Ocimene	13877-91-3	12.67	2.92	0.1–1.0	0.1–1.0
40	β-Cymene	535-77-3	13.17	2.9	0.1–1.0	>1.0
41	Terpinolene	586-62-9	13.5	3.26	>1.0	>1.0
42	Methyl 2-hexenoate	2396-77-2	13.75	2.48	0.1–1.0	0.02–0.1
43	*cis*-3-Hexenyl-1-acetate	3681-71-8	14.5	2.48	0.02–0.1	-
44	6-Methyl-5-hepten-2-one	110-93-0	15.08	2.38	-	0.1–1.0
45	1-Hexanol	111-27-3	15.42	1.88	0.1–1.0	0.02–0.1
46	3-Hexen-1-ol	544-12-7	16.25	1.84	0.1–1.0	<0.02
47	*trans*-2-Pentenol	1576-96-1	16.83	1.82	0.1–1.0	<0.02
48	2,5-Dimethyl-3-methylene-hepta-1,5-diene	74663-83-5	18.42	3.74	0.02–0.1	0.02–0.1
49	4-Thujanol	546-79-2	18.5	2.24	>1.0	0.1–1.0
50	γ-Elemene	29873-99-2	19.08	3.66	0.02–0.1	0.1–1.0
51	(−)-Camphor	464-48-2	20	2.7	0.02–0.1	-
52	(−)-β-Bourbonene	5208-59-3	20.08	3.78	0.02–0.1	0.02–0.1
53	Benzaldehyde	100-52-7	20.25	2.04	-	0.02–0.1
54	Linalool	78-70-6	20.58	2.06	0.1–1.0	0.1–1.0
55	*cis*-Sabinene hydrate	17699-16-0	20.67	2.16	>1.0	>1.0
56	*trans*-2-Menthenol	29803-81-4	21.08	2.18	0.1–1.0	0.1–1.0
57	Pinocarvone	30460-92-5	21.25	2.6	<0.02	0.02–0.1
58	Fenchol	1632-73-1	21.5	2.14	-	0.02–0.1
59	*trans*-Bornyl acetate	5655-61-8	21.5	2.96	0.1–1.0	0.02–0.1
60	β-Elemene	515-13-9	21.83	3.24	0.1–1.0	0.1–1.0
61	β-*cis*-Caryophyllene	118-65-0	21.92	3.64	0.02–0.1	0.1–1.0
62	Terpinen-4-ol	562-74-3	22	2.4	>1.0	0.1–1.0
63	Caryophyllene	87-44-5	22.08	3.6	0.1–1.0	>1.0
64	Patchoulane	25491-20-7	22.25	3.62	<0.02	0.02–0.1
65	Carvomenthenal	29548-14-9	22.42	2.54	0.02–0.1	0.02–0.1
66	*cis*-Menth-2-en-1-ol	29803-82-5	22.58	2.12	0.1–1.0	0.1–1.0
67	α-Gurjenene	489-40-7	22.58	3.82	<0.02	0.02–0.1
68	Aromandendrene	489-39-4	23.17	3.66	0.02–0.1	0.1–1.0
69	δ-Terpineol	7299-42-5	23.58	2.18	>1.0	>1.0
70	1,11-Dodecadiyne	20521-44-2	23.67	3.44	-	1.37
71	Humulene	6753-98-6	23.75	3.42	>1.0	0.1–1.0
72	*Z*,*Z*,*Z*-1,5,9,9-tetramethyl-1,4,7-Cycloundecatriene	-	23.75	3.44	-	0.1–1.0
73	2,5-Dihydrotoluene	4313-57-9	23.92	2.38	-	0.02–0.1
74	Neral	106-26-3	24	2.34	0.1–1.0	0.02–0.1
75	α-Terpineol	98-55-5	24.17	2.24	0.02–0.1	>1.0
76	(*+*)-α-Terpineol	7785-53-7	24.25	2.34	>1.0	>1.0
77	Alloaromadendrene	25246-27-9	24.33	3.52	0.02–0.1	0.1–1.0
78	1-(3-Methylenecyclopentyl)-ethanone	54829-98-0	24.42	2.1	-	0.02–0.1
79	Dodecanal	112-54-9	24.67	2.66	0.02–0.1	0.02–0.1
80	Germacrene D	23986-74-5	24.67	3.36	0.1–1.0	0.1–1.0
81	α-Phellandren-8-ol	1686-20-0	24.83	2.08	0.02–0.1	0.02–0.1
82	2-Hydroxycineol	18679-48-6	24.92	2.26	-	0.1–1.0
83	(−)-Lavandulyl acetate	20777-39-3	24.92	2.58	0.1–1.0	0.02–0.1
84	4-Methyl-2-pentene	4461-48-7	25.08	2.32	0.1–1.0	0.1–1.0
85	Bicyclogermacrene	24703-35-3	25.17	3.46	0.1–1.0	0.02–0.1
86	Germacrene B	15423-57-1	25.17	3.48	-	0.1–1.0
87	*trans*-Pipertiol	16721-39-4	25.25	2.1	0.1–1.0	0.1–1.0
88	α-Farnesene	502-61-4	25.42	3.04	0.02–0.1	0.02–0.1
89	(+)-δ-Cadinene	483-76-1	25.58	3.4	<0.02	0.02–0.1
90	Citronellol	106-22-9	25.67	2	<0.02	0.02–0.1
91	*cis*-Geraniol	106-25-2	26.42	2	0.1–1.0	0.1–1.0
92	Pentanoic acid	109-52-4	27.25	1.62	0.02–0.1	0.02 - 0.1
93	4-(2-Aetylamino-1-(acetyloxy)-ethyl)phenyl acetate	55044-38-7	27.42	1.96	0.1–1.0	0.1–1.0
94	Prenyl bromide	870-63-3	27.42	1.98	<0.02	0.02–0.1
95	Tetraethylene glycol	112-60-7	30.33	2.08	0.1–1.0	0.1–1.0
96	Caryophyllene oxide	1139-30-6	30.5	2.88	0.02–0.1	0.02–0.1
97	2-Methoxy-ethanol	109-86-4	30.58	2.06	0.02–0.1	0.02–0.1
98	Propylene glycol	57-55-6	31.08	2	0.02–0.1	0.1–1.0
99	Nerolidol	142-50-7	31.42	2.3	<0.02	0.02–0.1
100	Humulene epoxide II	19888-34-7	31.58	2.82	<0.02	0.02–0.1
101	Elemol	639-99-6	32.17	2.34	0.02–0.1	0.02–0.1
102	*trans*-*Z-*α-Bisabolene epoxide	-	32.17	2.5	-	0.02–0.1
103	Guaiol	489-86-1	32.33	2.48	0.1–1.0	0.1–1.0
104	Rosifoliol	63891-61-2	33	2.52	0.02–0.1	0.02–0.1
105	Spathulenol	6750-60-3	33.08	2.42	-	0.1 - 1.0
106	Triethylene glycol	112-27-6	33.17	1.86	0.1–1.0	0.02–0.1
107	Ethyl nitrosourethane	614-95-9	33.58	1.24	<0.02	0.02–0.1
108	1,2-Ethanediol	107-21-1	34.08	1.4	0.1–1.0	0.1–1.0
109	α-Dihydroionone	31499-72-6	33.42	2.42	0.02–0.1	0.1–1.0
110	Diethyl-mercury	627-44-1	34.5	1.24	-	0.02–0.1
111	Bulnesol	22451-73-6	34.58	2.48	0.02–0.1	<0.02
112	Icosa-9,11-diyne	28393-07-9	34.83	2.4	0.02–0.1	0.1–1.0
113	Paraldehyde	123-63-7	35.42	2.38	-	0.02–0.1
114	Limonene dioxide	96-08-2	35.42	2.4	0.02–0.1	-
115	16-Allopregnene-3β,9α-diol-20-one 3-*O*-acetate	106068-45-5	35.42	2.42	-	0.1–1.0
116	2-Hydrazinoethanol	109-84-2	36.25	1.72	-	0.1–1.0
117	Isoeugenol	97-54-1	36.83	1.92	0.02–0.1	<0.02
118	1-Bromo-2-propanol	19686-73-8	36.92	1.32	-	0.02–0.1
119	(1-Hydroxyethylidene)malonic acid diethyl ester	31575-84-5	37.25	1.26	>1.0	0.1–1.0
120	Diethylene glycol	111-46-6	37.25	3.36	0.1–1.0	0.1–1.0
121	Eicosane	112-95-8	38.08	3.78	0.1–1.0	-
122	Isopropyl alcohol	67-63-0	38.33	1.64	-	0.02–0.1
123	Pentaethylene glycol	4792-15-8	38.42	3.02	-	0.02–0.1
124	*R*-(−)-1,2-propanediol	4254-14-2	38.83	1.3	-	0.02–0.1
125	Norpseudoephedrine	36393-56-3	38.92	3.76	0.1–1.0	0.1–1.0
126	Metacetaldehyde	108-62-3	39	4.76	0.1–1.0	0.1–1.0
127	Ethyl2-(methoxyimino)acetoacetate	60846-14-2	39.08	1.6	-	0.1–1.0
128	Ethanolamine	141-43-5	39.42	1.32	>1.0	0.02–0.1
129	Erythro-3-bromo-2-pentanol	159475-12-4	39.5	1.42	0.1–1.0	0.02–0.1
130	Octaethylene glycol	5117-19-1	40.42	4.08	0.1–1.0	0.1–1.0
131	Dimethyl ethylboronate	7318-82-3	40.75	1.8	-	0.02–0.1
132	Cysteinylglycine	19246-18-5	41	1.74	0.02–0.1	-
133	Isophytol	505-32-8	41.33	2.46	0.02–0.1	0.02–0.1
134	2,5,8,11-Tetraoxadodecane	112-49-2	41.42	1.74	<0.02	0.02–0.1
135	2-ethoxy-propane	625-54-7	41.42	3.84	0.064	0.02–0.1
136	α-Methoxyacetaldehyde	10312-83-1	42.42	1.42	-	0.1–1.0
137	Diethylmethylsilane	760-32-7	42.5	2.4	0.1–1.0	0.02–0.1
138	1-Iodotetradecane	19218-94-1	43.08	4.62	0.02–0.1	-
139	1-Propoxy-2-propanol	1569-01-3	43.17	1.78	0.02–0.1	-
140	β-Nitroethanol	625-48-9	43.42	1.56	<0.02	0.1–1.0
141	Trimethylsilane	993-07-7	43.42	1.74	0.1–1.0	0.02–0.1
142	Carbitol	111-90-0	43.5	1.78	0.1–1.0	0.1–1.0
143	2-Methylamino-1-ethanol	109-83-1	43.92	3.74	-	0.02–0.1
144	3,6,9,12-Tetraoxatetradecan-1-ol	5650-20-4	43.92	3.8	0.1–1.0	0.1–1.0
145	Methoxytriglycol	112-35-6	44	2.28	0.02–0.1	-
146	Cysteine-alanine	-	44.08	1.74	0.02–0.1	0.02–0.1
147	Ethyltriglycol	112-50-5	44.17	3.78	0.02–0.1	<0.02
148	Dimethylethylhexadecylamm-onium bromide	124-03-8	44.33	1.26	<0.02	0.02–0.1
149	Formaldehyde	50-00-0	44.42	1.2	>1.0	>1.0
150	(3-Methyl-oxiran-2-yl)-methanol	-	44.42	1.3	>1.0	>1.0
151	Ethyldimethyl-silane	758-21-4	44.5	1.74	0.02–0.1	0.02–0.1
152	(2-Ethoxy-1-methoxyethoxy)-ethene	54063-18-2	44.58	2.28	-	0.02–0.1
153	(2-Pyrrol2-[2-(2-hydroxy-3-ethoxy)-ethoxy]-4-nitro-phenoxymorpho-ethoxy)-ethanol	-	44.75	2.54	-	0.1–1.0
154	3,4-Dimethyl-2-hexanone	19550-10-8	44.92	1.8	-	0.02–0.1
155	Pyruvaldehyde	78-98-8	45	1.76	0.1–1.0	0.1–1.0
156	2-(1-Ethoxyethoxy)-2-(2-oxiranyl)ethanol	109613-58-3	45.17	1.78	0.02–0.1	0.02–0.1
157	2-[2-(Ethenyloxy)ethoxy]-ethanol	929-37-3	45.42	1.86	>1.0	0.1–1.0
158	Isopropyldimethylsilane	18209-61-5	45.58	1.74	0.02–0.1	0.1–1.0
159	15-Crown-5	33100-27-5	45.75	3.86	-	0.1–1.0
	Total				>89.68	>87.88

^1^ t_R_, retention time in first dimension (min); ^2^ t_R_ (s), retention time in second dimension (s).

**Table 3:** The single factor test design.

Factors	Levels	Other Conditions
microwave power (W)	500, 600, 700, 800, 900	6:1 mL/g, 60 min
liquid-to-material ratio (mL/g)	2:1, 4:1, 6:1, 8:1, 10:1	optimized microwave power, 60 min
extraction time (min)	20, 30, 40, 50, 60	optimized microwave power and liquid-to-material ratio

**Table 4:** The factors and levels of the response surface methodology (RSM) design.

Factors	Codes	Levels				
		−1.68	−1	0	1	1.68
microwave power (W)	X_1_	631.82	700	800	900	968.18
liquid-to-material ratio (mL/g)	X_2_	1:2.64	4:1	6:1	8:1	9.36:1
extraction time (min)	X_3_	13.18	20	30	40	46.82

**Table 5:** GC×GC column sets.

	First Column	Second Column
stationary phase	DB-WAX	DB-17
dimension	30 m × 0.25 mm	1.7 m × 0.10 mm
film thickness	0.25 μm	0.10 μm
polarity	strong	medium
corporation	J&W Scientific, Folsom, CA	J&W Scientific, Folsom, CA
